# Nontraditional Electrocardiogram and Algorithms for Inconspicuous In-Home Monitoring: Comparative Study

**DOI:** 10.2196/mhealth.9604

**Published:** 2018-05-28

**Authors:** Nicholas J Conn, Karl Q Schwarz, David A Borkholder

**Affiliations:** ^1^ Microsystems Engineering Rochester Institute of Technology Rochester, NY United States; ^2^ School of Medicine and Dentistry University of Rochester Medical Center Rochester, NY United States

**Keywords:** algorithms, delineation, ECG, EDB, electrocardiogram, Internet of Things, IoT, MITDB, signal quality, wearable

## Abstract

**Background:**

Wearable and connected in-home medical devices are typically utilized in uncontrolled environments and often measure physiologic signals at suboptimal locations. Motion artifacts and reduced signal-to-noise ratio, compared with clinical grade equipment, results in a highly variable signal quality that can change significantly from moment to moment. The use of signal quality classification algorithms and robust feature delineation algorithms designed to achieve high accuracy on poor quality physiologic signals can prove beneficial in addressing concerns associated with measurement accuracy, confidence, and clinical validity.

**Objective:**

The objective of this study was to demonstrate the successful extraction of clinical grade measures using a custom signal quality classification algorithm for the rejection of poor-quality regions and a robust QRS delineation algorithm from a nonstandard electrocardiogram (ECG) integrated into a toilet seat; a device plagued by many of the same challenges as wearable technologies and other Internet of Things–based medical devices.

**Methods:**

The present algorithms were validated using a study of 25 normative subjects and 29 heart failure (HF) subjects. Measurements captured from a toilet seat-based buttocks electrocardiogram were compared with a simultaneously captured 12-lead clinical grade ECG. The ECG lead with the highest morphological correlation to buttocks electrocardiogram was used to determine the accuracy of the heart rate (HR), heart rate variability (HRV), which used the standard deviation of the normal-to-normal (SDNN) intervals between sinus beats, QRS duration, and the corrected QT interval (QT_c_). These algorithms were benchmarked using the MIT-BIH Arrhythmia Database (MITDB) and European ST-T Database (EDB), which are standardized databases commonly used to test QRS detection algorithms.

**Results:**

Clinical grade accuracy was achieved for all buttocks electrocardiogram measures compared with standard Lead II. For the normative cohort, the mean was −0.0 (SD 0.3) bpm (N=141 recordings) for HR accuracy and −1.0 (SD 3.4) ms for HRV (N=135). The QRS duration and the QT_c_ interval had an accuracy of −0.5 (SD 6.6) ms (N=85) and 14.5 (SD 11.1) ms (N=85), respectively. In the HF cohort, the accuracy for HR, HRV, QRS duration, and QT_c_ interval was 0.0 (SD 0.3) bpm (N=109), −6.6 (SD 13.2) ms (N=99), 2.9 (SD 11.5) ms (N=59), and 11.2 (SD 19.1) ms (N=58), respectively. When tested on MITDB and EDB, the algorithms presented herein had an overall sensitivity and positive predictive value of over 99.82% (N=900,059 total beats), which is comparable to best in-class algorithms tuned specifically for use with these databases.

**Conclusions:**

The present algorithmic approach to data analysis of noisy physiologic data was successfully demonstrated using a toilet seat-based ECG remote monitoring system. This approach to the analysis of physiologic data captured from wearable and connected devices has future potential to enable new types of monitoring devices, providing new insights through daily, inconspicuous in-home monitoring.

## Introduction

### Background

Wearable and internet-connected medical devices have the potential to fill a gap in patient monitoring, providing insights into disease progression and cardiovascular health between office visits, as well as enabling prevention-focused personalized care. The sluggish adoption of such technology within health care can be attributed to a lack of clinical value due to the large volume of difficult-to-interpret data and poor confidence in measurement accuracy [1,2]. This is due to use in uncontrolled environments and limited signal quality, resulting in noisy and highly variable signals that change from moment to moment. As a result, many wearable and connected devices do not meet the requirements for medical use and instead target self-management of fitness and well-being.

The literature approaches this problem by directly addressing noise in physiologic signals, through signal enhancement and noise removal techniques. A common approach to reproducing a clean signal (eg, photoplethysmogram) is to utilize information from other simultaneously gathered signals such as accelerometer data, impedance measurements, and/or multiple electrocardiogram (ECG) channels [3-5]. Additional statistical approaches utilize a priori information about the signal to de-noise signals for more accurate analysis [6-9]. Although these approaches can be beneficial, the assumptions made about the physiologic waveforms during this process have the potential to create false measurements, especially for those with cardiovascular disease who often have abnormal morphologies and rhythm [10].

An alternative approach to managing noise-corrupted physiologic signals is to reject regions that are insufficient for analysis, rather than forcing them through analysis. By automatically rejecting poor quality regions of physiologic signals, only regions where the subsequent algorithms can accurately determine specific measures are analyzed, increasing confidence. There are only a few examples in the literature that take this approach to rejecting poor-quality regions, where the focus is on hospital-grade medical equipment with the goal of reducing false alarms and increasing the confidence of automated results [11,12], or where results are only presented for healthy subjects [13,14]. Since these algorithms have been designed for hospital-grade equipment used in a controlled environment or on normative subjects, they have limited applicability to wearables or in-home devices that typically contain greater variability in signal quality.

This study expands upon the literature by implementing a robust signal quality classification algorithm for the rejection of poor-quality regions designed specifically to work with a highly accurate delineation algorithm designed for noisy signals. The effectiveness of these algorithms is demonstrated using a nonstandard, dry electrode–based ECG integrated into a toilet seat for inconspicuous in-home monitoring. The objective of this work was to demonstrate successful utilization of this approach to physiologic analysis through a study of both normative and HF subjects.

### Motivation for In-Home Electrocardiogram Monitoring of Heart Failure

Heart failure (HF) occurs when the heart muscle is weakened and unable to maintain the blood flow required to meet the body’s needs. Approximately 6.5 million Americans have HF, with 960,000 new cases per year [15]. HF costs the United States an estimated US $30.7 billion each year and is expected to increase to 127% to reach $69.7 billion by 2030 [15]. With approximately 80% of the total cost associated with HF due to hospitalization [16], there is an opportunity to reduce the cost of HF by lowering hospitalization rates through remote patient monitoring. The literature shows that hospital readmissions of HF patients can be reduced by the remote monitoring of a single-lead ECG [17-19]. Although a single lead has limited diagnostic capabilities, it can be useful for monitoring of disease progression, specifically through tracking arrhythmias, heart rate (HR), heart rate variability (HRV), QRS duration, and corrected QT interval (QT_c_).

Changes in both HR and HRV can be used to predict cardiovascular events. For individuals with or without coronary artery disease, resting HR is a predictor of mortality, independent of other risk factors [20,21]. Low HRV is associated with a 32% to 45% increased risk of a first cardiovascular event for patients with no previous history of cardiovascular disease [22]. It is also associated with chronic HF, diabetes, and alcoholic cardiomyopathy [23].

An increase in QRS duration can be used in the diagnosis of disease state and as a predictor of sudden death [24]. For example, a QRS width of greater than 120 ms suggests that cardiac dyssynchrony may be present [25]. In addition, QRS duration may have secondary value in predicting the prognosis of patients with HF [24]. In one study, implantable cardiac defibrillator patients with HF who had a wide underlying QRS complex showed more than double the rate of cardiac mortality than those with a narrow QRS complex [26]. The degree of QRS prolongation is correlated with an increase in severity of left ventricular systolic dysfunction, left ventricular dilation, and mitral regurgitation [27]. Left ventricular function worsens as the QRS duration increases [26-29], making it an important parameter to monitor over time.

A prolonged QT_c_ interval is a strong, independent predictor of adverse outcomes in patients with HF, because it is related to ventricular polarization and repolarization [30]. Many drugs prescribed to cardiovascular patients change the PR interval and the QRS duration. However, they can also prolong the QT interval, which can be very dangerous. A drug-induced prolongation of the QT interval is associated with Torsades de Pointes (a polymorphic ventricular tachycardia), which may cause sudden cardiac death (unexpected cardiovascular collapse without warning) [31,32].

### The Opportunities and Challenges of In-Home Electrocardiogram Monitoring

Currently, ECG monitoring is performed in a hospital or doctor’s office, or for a short duration (typically from 1 to 7 days) at a patient’s home using a Holter monitor [33,34]. Daily monitoring has the potential to avoid issues with the episodic nature of hospital or doctor visits and to provide insights beyond short-term Holter monitoring. Visits to the hospital or doctor occur neither at a consistent time of day nor with the subject at a consistent physiologic state, making it difficult for physicians to see trends in the measured parameters across time. In addition, white coat syndrome can significantly affect measurement results.

Although the 12-lead ECG cannot be replaced with a single-lead ECG for diagnostic purposes, there is an opportunity to fill a gap in patient monitoring with daily single-lead ECG measurements. If data can be gathered reliably, physicians can begin using each of these parameters (HR, HRV, QRS duration, and QT interval) to monitor disease progression over time. This would allow trends to be picked up that would otherwise be missed, enabling an alert-based system for facilitating early intervention. The many challenges of in-home physiological monitoring that are not present in a hospital environment or doctor’s office need to be addressed for the data to be gathered reliably. In the home, a trained expert is not on hand to make any real-time changes that would ensure correct electrode placement and signal integrity. In addition, patient compliance is often low, resulting in inconsistent data collection that impedes accurate trend analysis. The fully integrated toilet seat form factor and the algorithms presented herein have the potential to address many of these challenges.

### A Toilet Seat–Based Electrocardiogram Addresses Challenges in Patient Compliance for In-Home Monitoring

A toilet seat–based cardiovascular monitoring system can be integrated into a subject’s natural daily routine with no change in habit, enabling measurements to be taken at one or more times each day. Furthermore, issues with subject preparation and subject error are greatly reduced, since skin contact is automatic and has sufficient pressure to create a repeatable electrode interface at a consistent location for each subject. Although a toilet seat**–**based buttocks electrocardiogram (bECG) is intermittent in nature, ensured compliance will enable long-term daily tend monitoring of parameters that do not require continuous monitoring, such as the QRS duration and QT_c_ interval.

### Challenges Associated With a Toilet Seat–Based Electrocardiogram

Standard gel-based ECG electrodes cannot be used in a toilet seat-based device, necessitating the use of dry electrodes. Both the measurement location and the use of dry electrodes increase the noise present in the captured signal and reduce the amplitude of the ECG signal. Despite these challenges, the literature shows that it is possible to capture an ECG from a toilet seat [35-40]. Only the work presented in by Baek et al [38] has quantitatively compared the ECG from a toilet seat to a gold-standard ECG measure, where capacitive electrodes on the seat and wet electrodes placed adjacent on the thigh were compared with a standard limb lead ECG. The results of this study showed that manual R-peak delineation resulted in less than a 2-ms error in location and that the estimated HR was within 0.003 bpm for a single test subject. To date, no study has quantitatively compared the bECG HRV, QRS duration, QT_c_ interval, and waveform morphology with a clinical 12-lead ECG.

Unique algorithms have been developed to address the challenges associated with capturing and analyzing the bECG. The broad objective of this work was to demonstrate the feasibility of the present algorithms and bECG-based system for accurately monitoring key cardiovascular parameters in both a healthy population and an HF population, as a precursor to long-term trend-based intervention studies. The human subject data used herein were obtained at the Rochester Institute of Technology and the University of Rochester Medical Center. Studies were performed under informed consent and used protocols approved by each institution’s Institutional Review Board for Protection of Human Subjects. These controlled studies compare the capabilities of the bECG to a clinical grade 12-lead ECG, quantitatively comparing the accuracy of extracted R-peaks, HR, HRV, waveform morphology, QRS duration, and QT_c_ interval for algorithm validation.

## Methods

### A Toilet Seat–Based Buttocks Electrocardiogram

**Figure 1:**
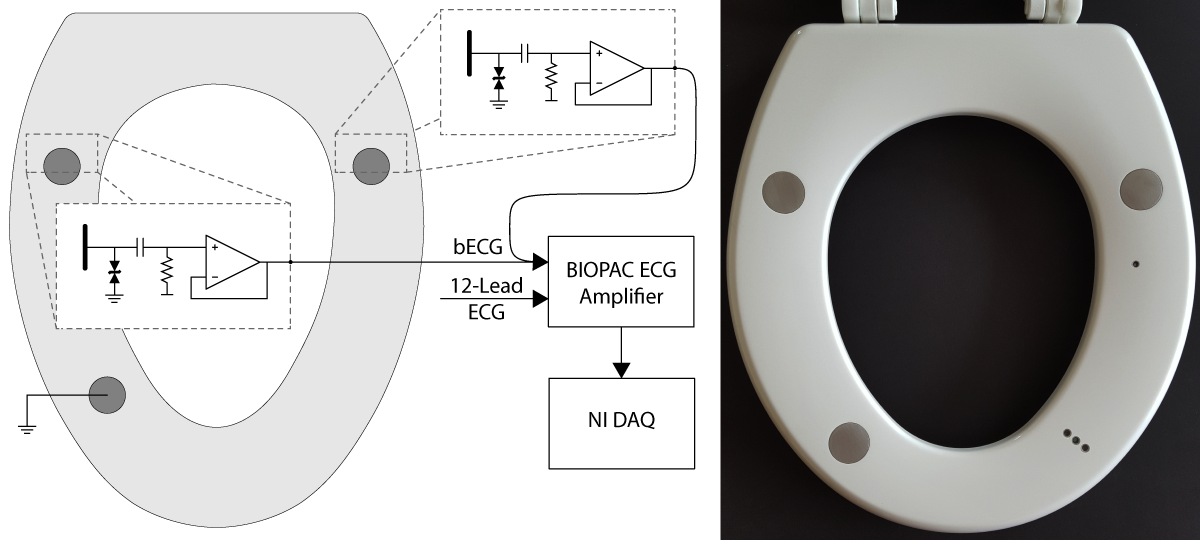
Stainless-steel electrodes are integrated into the seat and are connected directly to the integrated ECG analog front-end. The output of each front-end is low-impedance, allowing for a low-noise connection to the differential ECG amplifier inside of the BIOPAC MP150 system (left). The resulting full-scale ECG signal is acquired on a NI CompactRIO DAQ. For the HF subject study, all instrumentation and data acquisition circuitry are integrated into the toilet seat (right). DAQ: data acquisition system; ECG: electrocardiogram; bECG: buttocks ECG; NI: National Instruments; HF: heart failure.

The bECG is integrated into an elongated toilet seat, with dry electrodes on the surface and electronic instrumentation inside of the seat (Figure 1). It contains three electrodes, consisting of a differential electrode pair and grounded right leg reference, each with a diameter of 28 mm. Stainless-steel electrodes are chosen for their noncorrosive and nonirritant properties. The differential electrode pair is placed on the seat such that skin contact is made in proximity to the subject’s gluteal fold when seated. A grounded right leg electrode is placed approximately 12 cm below the differential electrode pair on the right side of the toilet seat from the vantage point of a seated subject (Figure 1). Each electrode is securely integrated into the surface of the toilet seat with epoxy, to ensure repeatability across recordings.

The active front-end instrumentation is integrated inside of the seat and connected to each of the differential stainless-steel electrodes with welded wires. This results in a maximum distance of 10 mm between the electrode and the front-end instrumentation. The active electrodes contain electrostatic discharge protection and a high-pass filter with a −3 dB cutoff frequency of 0.16 Hz that removes any direct current voltage bias present on the body. This ensures that the signal is within the valid input voltage range.

For the normative subject study, the instrumentation is powered by a 3.3 V boost converter, which in turn is powered by a 3.7 V (nominal voltage) rechargeable lithium polymer battery. The output from each active electrode is differentially amplified using the ECG instrumentation (ECG100C) within a BIOPAC MP150 system (BIOPAC Systems, Inc, Goleta, CA, USA), which is also used to gather a 12-lead gold standard ECG. All signals are then acquired with a sample rate of 1000 Hz using a National Instruments cRIO-9075 CompactRIO Data Acquisition System (National Instruments Corp., Austin, TX, USA), which is controlled by a laptop. The toilet seat with integrated electrodes and active ECG front-end instrumentation is secured to an elongated toilet mounted to the floor. A schematic overview of the system is shown in Figure 1. All devices that are not battery-powered are plugged into a medical-grade isolation transformer (ILC-1400MED4) to ensure electrical safety (TSi Power Corp., Antigo, WI, USA).

For the in-hospital HF subject study, all instrumentation and data acquisition circuitry are integrated into the toilet seat (Figure 1). The instrumentation is powered by a 3 V linear regulator that is powered by a 3.3 V boost converter, which in turn is powered by a 3 V primary lithium battery. The AD8232 (Analog Devices, Inc, Norwood, MA, USA) is used to differentially amplify the bECG in the cardiac monitor configuration application circuit [41]. The integrated ADC in the MSP430FR5969 (Texas Instruments, Inc, Dallas, TX, USA) is used to acquire the bECG signal, which is sent through a universal serial bus to a laptop computer. The 12-lead ECG is acquired using the BIOPAC. A pulsatile transistor-transistor logic signal sent from the MSP430FR5969 and captured with the BIOPAC data channels enable both systems to be synchronized automatically, with a timing offset error no greater than ±1 ms. All devices that are not battery-powered are plugged into a medical-grade isolation transformer (ILC-1400MED4) to ensure electrical safety (TSi Power Corp., Antigo, WI, USA).

### Normative Subject Testing

Normative subject testing was performed on 26 healthy subjects who had no history of heart disease. One subject was rejected from inclusion in the normative study due to a prolonged QRS duration and abnormal ECG morphology in the 12-lead ECG, as determined by a Board-Certified cardiologist. Of the remaining 25 subjects, there were a total of 13 male and 12 female subjects in the age group of 20 to 50 years, with a mean age of 26.7 years. For the at-rest measurements, five 150-s (2.5-min) recordings were captured with each subject sitting on the toilet seat at rest. Between recordings, each subject was instructed to stand up to introduce positioning differences that would normally be associated with multiple uses of a toilet in the home, potentially changing signal quality and waveform characteristics. Next, to induce stress, each subject was instructed to raise his or her HR to 75% of their maximum predicted HR (220 – age) on a Schwinn 230 recumbent bicycle and then quickly transition back to the toilet seat upon reaching the desired HR. For the poststress measurement, a final 150-s (2.5-min) recording was then taken for each subject. A simultaneous, standard, 12-lead ECG with gel electrodes was acquired using the ECG100C ECG amplifier and MP150 system from BIOPAC.

### Heart Failure Subject Testing

Testing was performed on 29 subjects diagnosed with HF in a hospital setting; there were a total of 21 male and 8 female subjects in the age group of 22 to 83 years, with a mean age of 55.4 years and a body mass index of 29.9 (SD 7.7) kg/m^2^. At the time of testing, all the subjects were inpatients due to HF. Seven 150-s (2.5-min) recordings were captured with each subject sitting on the toilet seat at rest. Between recordings, each subject was instructed to stand up to introduce positioning differences that would normally be associated with multiple uses of a toilet in the home, potentially changing signal quality and waveform characteristics. Measurements were only gathered at rest from the HF subjects. A simultaneous, standard, 12-lead ECG with gel electrodes was acquired using the ECG100C ECG amplifier and MP150 system from BIOPAC.

**Figure 2:**
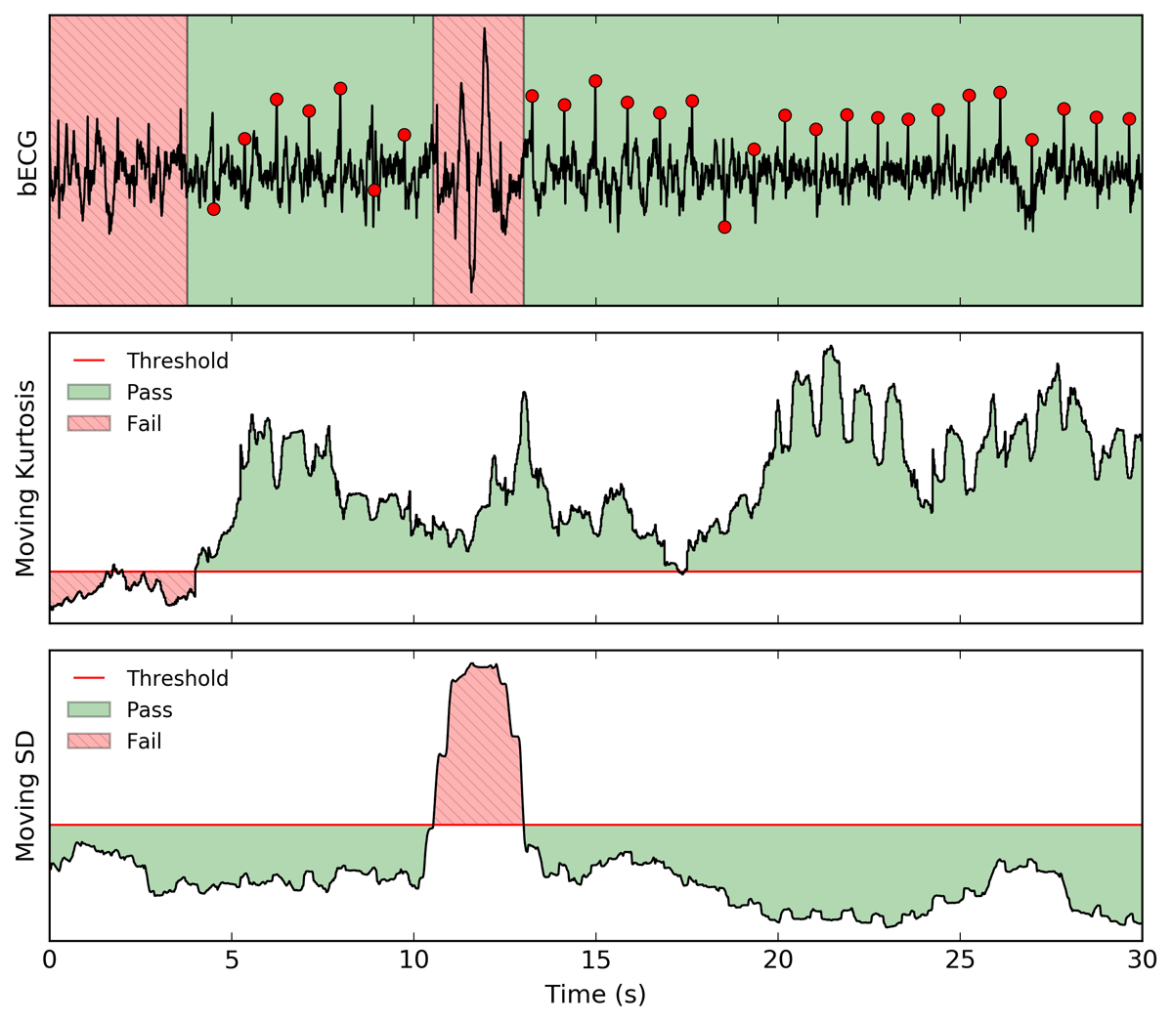
Signal quality is calculated from the bandpass filtered (1-45 Hz) and notch filtered (60 Hz) bECG (top) using a moving kurtosis (middle) and moving SD (bottom). The first signal quality index (moving kurtosis) rejects regions with in-band noise that have a kurtosis below a threshold of 3.6. The second signal quality index (moving SD) rejects noisy spikes that have a moving SD greater than 1.25 times the overall SD. R-peak delineations are shown as red circles to indicate where each beat is located within regions that have passed the signal quality test. bECG: buttocks electrocardiogram.

### Automated Signal Quality Classification

Rejecting regions of poor signal quality is necessary for the accurate analysis of the dry electrode bECG because it is more prone to noise and motion artifacts than traditional wet electrode ECG systems. Custom algorithms were developed to automatically assess signal quality and reject noisy waveform segments using two signal quality indices (SQIs): one based on the kurtosis and the second based on the SD for spike detection.

The kurtosis is a statistical measure that is commonly used to determine ECG signal quality [42-44]. It is defined as the fourth moment about the mean (*µ*_4_), divided by the SD to the fourth power (*σ*^4^), as shown in equation 1. The kurtosis is a statistical measure of the *tailedness* of a distribution, where a normal distribution has a kurtosis of 3. When the kurtosis is lower than 3, the distribution under test has longer tails than a normal distribution. Typically, the kurtosis is calculated across a large window of at least 10 s and is used to locate large motion artifacts or excessive baseline wander [42]. A clean, sinus rhythm ECG with no motion artifacts or baseline wander has a kurtosis of greater than 5 [45].

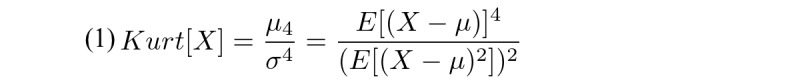



Here, to identify waveform segments that contain excessive in-band noise, the moving kurtosis is calculated across a 2-s window on a bandpass-filtered ECG with a bandwidth of 5 to 15 Hz (second-order Butterworth filter), chosen to isolate the QRS complex. By using a smaller 2-s window, the kurtosis measure is no longer dominated by episodic large motion artifact or baseline wander. A kurtosis threshold of 3.6 was empirically chosen for this work based on the normative subject data. An example of the resulting kurtosis value compared with the threshold of 3.6 for a typical waveform is shown in Figure 2.

Large-amplitude spikes due to motion artifacts are detected using a second-stage SQI. Spikes are identified as an increase in the moving SD within a 2-s window. In this new approach, a threshold of 1.5 times the SD of the entire signal was empirically determined to provide robust rejection of noisy spikes. Figure 2 shows an example of the resulting SQI and threshold.

### R-Peak Delineation Using a Modified Version of the Pan–Tompkins Algorithm

**Figure 3:**
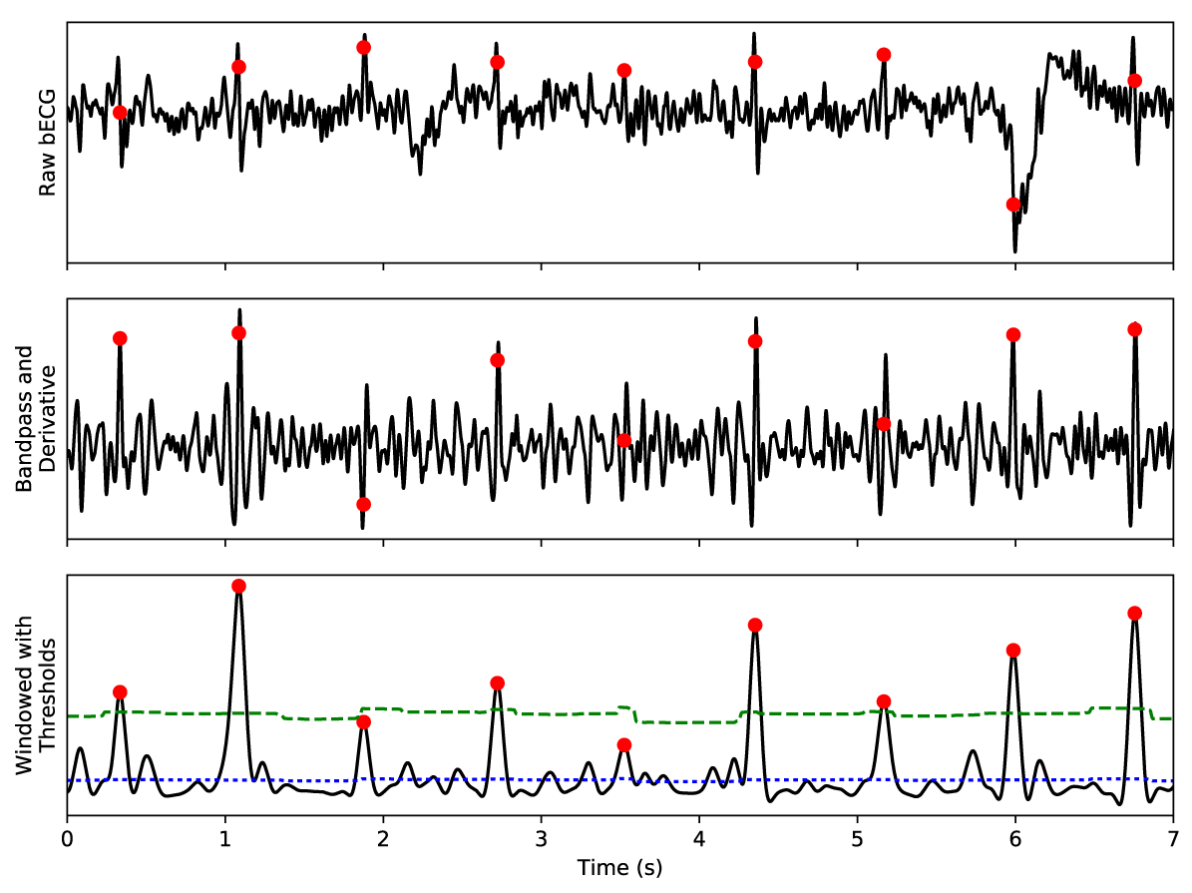
The modified PT algorithm processes the raw ECG signal (top) to isolate QRS complexes for delineation using a bandpass filter and derivative (middle). Dual thresholds (dashed and dotted lines) are calculated using a moving window average and are continually updated (bottom), rather than updating only when a delineated feature is found. The resultant delineations, shown as red circles, are often shifted from the R-peak location, necessitating a subsequent refinement stage. ECG: electrocardiogram; PT: Pan–Tompkins.

ECG R-peaks are delineated on a beat-by-beat basis using a modified version of the well-known Pan–Tompkins (PT) algorithm [46]. The raw ECG signal is processed to isolate the QRS complex in both the frequency domain and the temporal domain. A first-order Butterworth bandpass filter with a bandwidth of 5 to 15 Hz is used on the input ECG waveform. This is the same filter that was used in the signal quality algorithm. A five-point derivative of the filtered ECG is taken and the resulting waveform is then squared, as described in the original PT algorithm [46]. The squared signal is low-pass filtered using a third-order Butterworth filter with a −3 dB cutoff frequency of 8 Hz, such that the resulting waveform will have smooth, individual peaks for each QRS complex. The cutoff frequency of 8 Hz was chosen to match the duration of a prolonged QRS complex, which is 120 ms [25,27]. Two thresholds are calculated from the resulting signal using a 5-s moving average (chosen to include multiple beats regardless of the HR), where the upper threshold is 1.25 times the moving average and the lower threshold is 0.3 times the moving average. Example waveforms from various stages of the modified PT algorithm are shown in (Figure 3), including both thresholds.

Each peak in the processed signal is located from largest-to-smallest amplitude with a minimum duration of 200 ms between peaks, corresponding with the cardiac refractory period [46]. Peaks that have an amplitude greater than the larger threshold are delineated. The median beat interval is calculated using all beats within 20 s of the current interval under test, to allow for natural variations in HR over time. If any of the resulting beat intervals are greater than 1.5 times the median beat interval, the lower threshold is used to locate missing peaks.

This algorithm differs from the original PT algorithm in Pan et al [46], which was designed to use integer arithmetic for real-time functionality on an 8-bit embedded system. Specifically, the present algorithm uses alternate filtering approaches and a different mechanism for calculating the dual thresholds. A Butterworth filter with a bandwidth that better isolates the constituent frequencies of the QRS complex is used, and a third-order low-pass filter is used in place of moving window integration. The modified PT thresholds are continuously updated to incorporate magnitude information from the peak, baseline noise, motion artifacts, and other ECG features to dynamically adjust to extreme changes in signal quality typical of dry electrodes.

Locating the exact peak of the R-wave is necessary when ensemble averaging to avoid feature smearing. Both the PT algorithm and the modified PT algorithm do not always locate the exact peak of the R-wave because the exact location is smoothed out by the processing stages, as shown in Figure 3. The R-peak location is refined by locating the two largest peaks in the squared derivative of the filtered ECG signal, within 150 ms of the original delineated point. These two peaks bound the search window for the R-peak, which is defined as the first zero-crossing of the first derivative of the filtered ECG signal.

The modified PT algorithm with signal quality-based classification is verified using the annotated MIT-BIH Arrhythmia Database (MITDB) and European ST-T Database (EDB) as a gold-standard [47,48]. The purpose of using both databases is to enable a direct comparison between the present algorithm and other published QRS delineation algorithms and to demonstrate that the algorithm performs well on a standard ECG. The MITDB contains 48 records that are each 30 min in duration, and the EDB contains 90 records that are each 120 min in duration, provided by PhysioNet [49]. The signals from the MITDB and EDB have not been resampled because the modified PT algorithm does not require the ECG to be a specific sample rate. The beat-by-beat (bxb) function in the WaveForm DataBase application [49] was used to determine the sensitivity (Se) and positive predictive value (PPV) of the algorithm compared with the gold-standard annotations using a standard acceptance window (eg, match window) of 150 ms. The aforementioned signal quality algorithm was used to indicate periods of shutdown (regions of poor signal quality), where the classification results are tallied separately. The beats that would be missed during the shutdown period are excluded when calculating the number of false negatives (FN). In addition, the original PT algorithm has been implemented as described in Pan et al [46] and tested on both standard databases and the bECG dataset to facilitate a direct comparison with the modified PT algorithm.

### Ensemble Averaging

**Figure 4:**
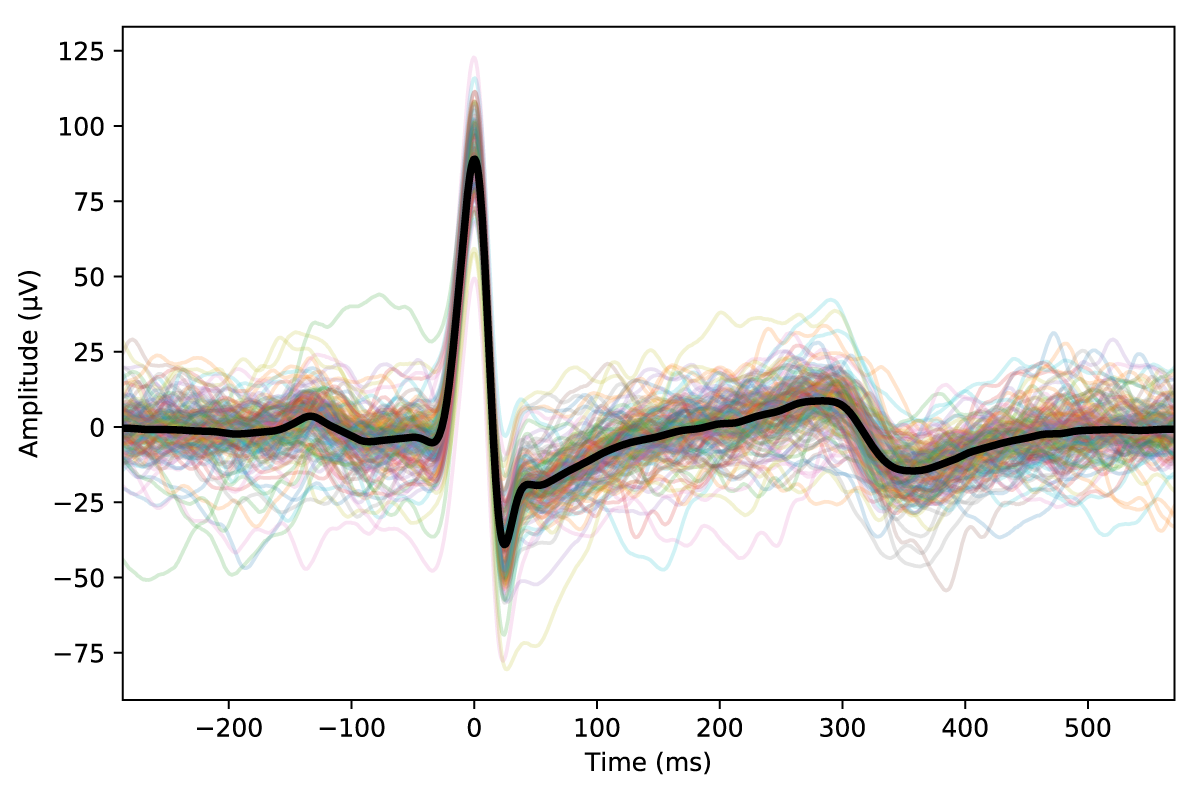
Ensemble averaging aligns multiple beats based on the R-wave peak location to calculate an average beat. Individual beats that passed all the rejection criteria are shown as thin light-colored lines, and the resulting ensemble averaged beat is shown as a thick dark line.

Ensemble averaging is a technique used in ECG signal processing to reduce noise and improve feature prominence within the cardiac cycle. Features such as the T-wave may not be visible on a beat-by-beat basis, but ensemble averaging allows these features to become clear and easily located. This study uses standard ensemble averaging techniques [50,51], in which each beat is stacked relative to the R-peak and then averaged sample-by-sample as shown in Figure 4. Only sections of the ECG that pass the signal quality index algorithm and two additional ensemble average beat rejection criteria are included when generating the ensemble average. The first additional criterion for rejection is based on the beat interval; only beats within ±10% of the median HR are included. The second criterion is that the root mean square (RMS) of the beat under test must be between 75% and 200% of the median RMS of every delineated beat.

This process is required for accurate delineation of the Q-wave onset, S-wave end, and T-wave end when analyzing an ECG captured using dry ECG electrodes. Despite the benefit of using ensemble averaging, this process removes beat-to-beat variations such as T-wave alternans. This type of analysis, including arrhythmia analysis, must be performed separately before ensemble averaging.

### Q-Wave, S-Wave, and T-Wave Delineation

Once the ensemble averaged beat is generated, the Q-wave onset, S-wave end, and T-wave end become clearly visible, allowing manual delineation of each feature. A trained expert manually located each of these three features using a custom graphical interface. Manual delineations were only used to calculate the QRS duration and QT interval for each recording. Delineations were spot-checked by 2 additional independently trained experts to ensure features were delineated correctly. Recordings where features are not clearly visible are marked as insufficient quality and are not included in the QRS or QT analysis by the trained expert. The bECG channel is delineated first so that the trained expert is not biased by prior knowledge of the gold-standard lead. After the bECG is completely delineated, the gold-standard lead undergoes the same delineation process.

The QRS duration is calculated as the time between the Q-wave onset and S-wave end. The Q-wave onset is defined as the return to baseline before the Q-wave. If no Q-wave is visible, it is defined as the initial deviation of the QRS complex from baseline. The S-wave end is defined as the point of inflection after the S-wave before the T-wave or before the return to baseline. If no S-wave is visible, the final return of the QRS complex to baseline is used. For the purposes of this work, the T-wave end is defined as the return to baseline after the maximum point of the downslope of the T-wave (or upslope, in the case of an inverted T-wave). The corresponding uncorrected QT interval is defined as the interval between the Q-wave onset and the T-wave end.

### Correlation Analysis to Select a Gold-Standard Electrocardiogram Lead

To determine the accuracy of the bECG QT_c_ interval and QRS duration, a standard ECG lead is required as a gold-standard. Each of the 12 standard leads is a projection of the heart dipole and is unique in shape. As the heart dipole changes magnitude and orientation during the cardiac cycle, ECG features measured on each differential pair have different timing, amplitude, and orientation. Differences in projection affect the extracted cardiovascular intervals (eg, bias in the QT_c_ interval) and the clinical interpretation of any given ECG lead [52]. The bECG is a nonstandard lead, so correlation analysis was used to determine which of the 12 standard leads most closely matched the bECG morphology. Analysis was performed on the ensemble averaged beats of each lead from each normative recording. To minimize errors that are introduced by timing differences in the R-peak locations, R-peaks from a single channel were used across all 12 leads and the bECG. This ensured a consistent reference point when ensemble averaging. The R-peaks from chest lead V1 were selected as a reference for all other leads, because this lead contained the fewest motion artifacts.

Pearson correlation coefficient, which is a measure of the linear correlation between two variables, was used to determine how closely each standard lead was to the bECG. This measure is calculated by dividing the covariance of the two variables by the product of their SD. Resulting values are between −1 and +1 (where *−1* represents a perfect negative linear relationship and *+1* represents a perfect positive linear relationship). Tukey boxplots were generated across the normative subject dataset, with whiskers 1.5 times the interquartile range to show the range of correlations between each lead. A paired Student *t*-test was used to determine if there was a statistically significant difference in the correlation results for each of the standard leads.

### Beat Classification Analysis

The efficacy of the bECG was evaluated by comparing clinically relevant parameters to those extracted from the highest correlated limb lead, as determined in the Methods section. Beat classification is used to determine how consistently and accurately beats can be located on the bECG. The bECG signal quality must be sufficient for robust determination of beats; otherwise, it cannot be used for further analysis. Six recordings from each normative subject were used in these analyses: five at rest, and one post stress.

The Se and PPV (also known as precision) were then calculated for the bECG waveform using the corresponding beat delineations from the gold-standard ECG channel. Feature locations identified within an acceptance interval on either side of the gold standard were considered true positives (TPs), while reported time indices that are not within this acceptance interval were considered false positives (FPs). If a corresponding feature was not found within the acceptance interval of the gold-standard feature, it was considered a missed beat, or an FN.

The interval used when analyzing standard databases is 150 ms, based upon the bxb function provided by PhysioNet [49]. For normative analysis a more stringent acceptance interval of 100 ms was chosen, as it is half the myocardium refractory period of 200 ms [46]. This ensures that multiple beats will not be present for a single gold standard beat within the acceptance window.

The Se, representing the percentage of correctly delineated beats, is calculated using equation 2:

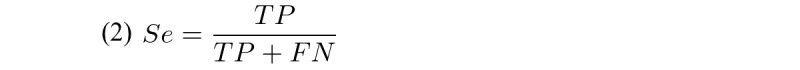



The PPV, representing the probability of a detected time index being a true positive, is calculated using equation 3:

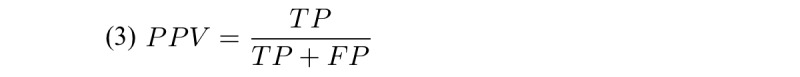



### Cardiac Intervals Analysis

For each recording the HR, HRV, QRS duration, and QT_c_ interval measured from the bECG were compared with the gold-standard lead that had the highest correlation with the bECG. The HR for a specific recording was calculated by taking the median RR interval (interval between consecutive R-peaks) after rejecting RR intervals that were more than 60% out of tolerance with respect to the initial median RR interval. Multiple methods can be used to calculate HRV. In this study, the SD of the RR intervals (SDNN) was used. For HR and HRV a minimum of 15 and 60 beats per recordings were required for analysis, respectively; if fewer beats were present, the recording was rejected and not included in the results.

QT_c_ and QRS intervals were extracted from the manually delineated feature timing on the ensemble averaged waveform for both the bECG and the gold-standard ECG lead. The minimum number of beats required for generating the ensemble averaged waveform for QT and QRS analysis was 60 beats. If fewer beats were present, the recording was rejected and not included in the analysis. Results were compared using Bland–Altman plots. Correcting the QT interval for different HRs is necessary when looking for trends or comparing across recordings. The QT_c_ interval is calculated using Bazett’s formula [53], which is as follows in equation 4:

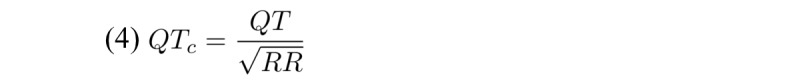



## Results

### The Buttocks Electrocardiogram Is Closely Correlated With Standard Electrocardiogram Limb Lead II

**Figure 5:**
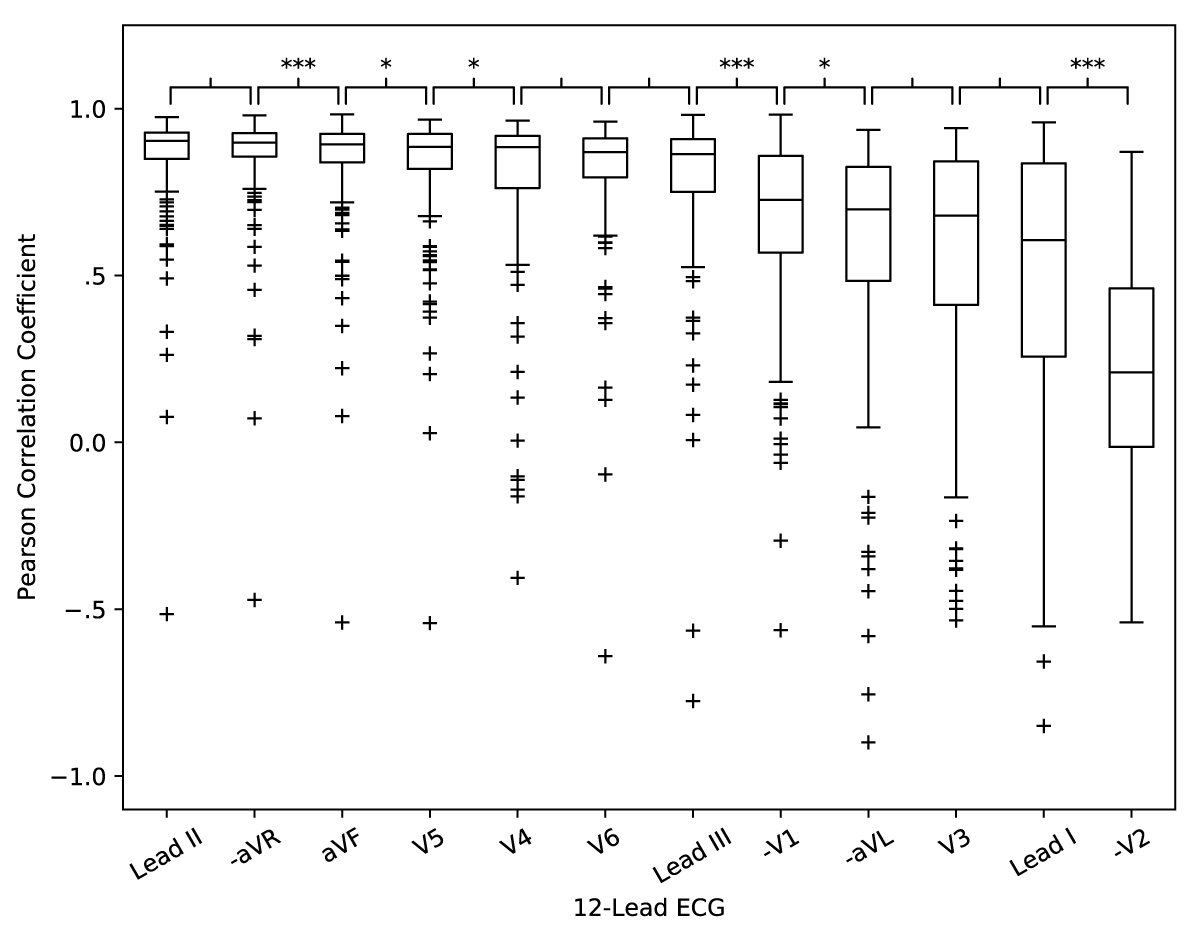
Box plots across normative recordings (N=140) show the correlation between the bECG and the standard ECG leads. Leads aVR, V1, aVL, and V2 have been inverted to facilitate visual comparison. Leads are organized from left to right by highest to lowest correlation. The top 3 correlated leads were Lead II, aVR, and aVF with median correlations of .904, .899, and .893, respectively. Statistical significance was tested using a paired Student t-test (*P<.05, **P<.01, and ***P<.001). bECG: buttocks electrocardiogram, ECG: electrocardiogram.

Correlation results between the bECG and each of the standard 12-leads are shown in (Figure 5), where the leads are shown in the order of highest to lowest median correlation. The bECG had the highest correlation with Lead II, *−* aVR, and aVF, with median correlations of .904, .899, and .893, respectively. The correlations for aVR, V1, aVL, and V2 have been inverted to facilitate visual comparison for high negative correlation. While the R-peaks from V1 were used as reference points for the ensemble averaging in the correlation analysis, there is no bias toward V1 since ensemble averaging is a linear process and every ensemble averaged beat from each of the 12 leads used the same reference points. Because each of the 12 leads was time-synchronized, a paired Student *t*-test was used to test whether differences in calculated lead correlations were statistically significant (Figure 5). Although there was no statistically significant difference between Lead II and −aVR (*P*=.41), Lead II was chosen as the gold-standard for the bECG since −aVR is an augmented lead calculated from Lead II.

### Successful Validation on Standard Databases

The algorithms presented herein have been tested on the MITDB and the EDB to evaluate their accuracy against established standards. Both the MITDB and EDB are standard databases that contain hand-annotated gold-standard references. These databases are commonly used to verify beat delineation algorithms. The Se, PPV, and accepted signal quality percentages for the MITDB and EDB are shown in (Table 1). The algorithms presented herein have a cumulative Se of 99.83% and PPV of 99.82% across both databases, while the original PT algorithm has a cumulative Se of 99.72% and PPV of 95.93%. The slight differences in classification results for the original PT algorithm compared with the published results, which were only provided for the MITDB (Se=99.75% and PPV=99.54%) [46], can be attributed to minor difference due to incomplete implementation details in the original work. The modified PT results are comparable with best-in-class algorithms that typically have an Se and PPV over 99.5%, with very few over 99.8% [54]. The cumulative statistics were generated by calculating the total number of TP, FN, and FP across both the MITDB and the EDB.

### Buttocks Electrocardiogram Delineations Robustly Correlate to Gold-Standard Lead II

**Figure 6:**
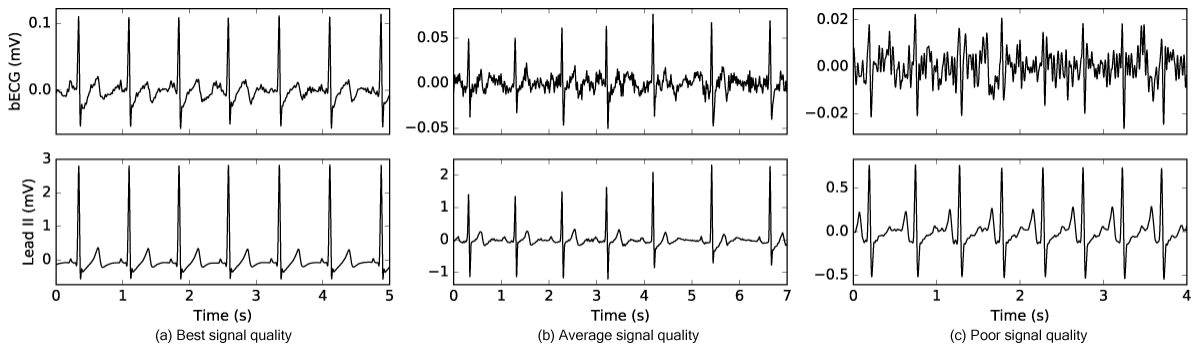
The signal quality of the bECG can vary across subjects and measurements because it is captured using active, dry electrodes. Three examples of signal quality are shown for the bECG (top) compared with a time synchronized Lead II ECG (bottom). The best signal quality example (a) shows the best signal quality achieved from the bECG study. The average example quality (b) shows the typical signal quality of the bECG. The poor example (c) shows a very noisy waveform that passed the signal quality check before analysis. bECG: buttocks electrocardiogram; ECG: electrocardiogram.

**Figure 7:**
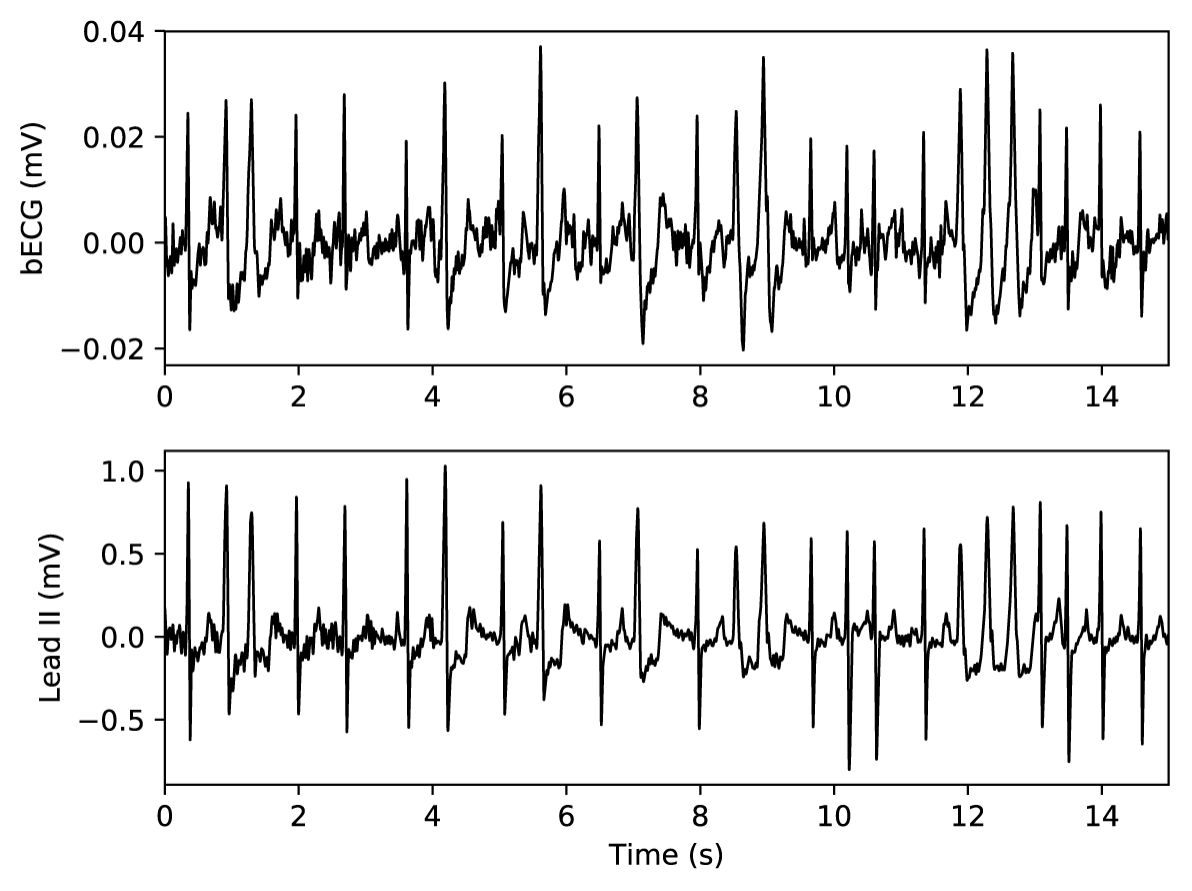
An example waveform from a heart failure subject during a period of arrhythmia, demonstrates that the bECG has sufficient quality to perform single-lead based rhythm analysis for those with disease states. bECG: buttocks electrocardiogram.

One fundamental difference between the standard clinical ECG and the bECG is the signal amplitude. Both the electrode location relative to the heart dipole and the type of electrode can reduce signal amplitude. Example bECG waveforms that have been preprocessed with a bandpass filter (1-45 Hz) and notch filter (60 Hz) for visualization are shown in Figure 6, illustrating best, average, and poor signal quality compared with a time-synchronized Lead II. Each of these waveforms has passed the SQI test and was considered to have sufficient quality for analysis. Effective signal quality utilization, as well as the use of robust algorithms, are absolute requirements in this application, because the bECG is acquired using dry electrodes and is much more prone to noise and motion artifacts than a typical wet electrode ECG. This is of additional importance for HF subjects, where ECG amplitude is often low compared with normative subjects due to myocardial necrosis. Despite these challenges the bECG captures rhythm and critical waveform features in the HF population, as shown in Figure 7.

Across 54 normative and HF subjects with a total of 882.5 min of data, 60.1% (530.4 min) of the bECG signal passed the signal quality algorithm with an overall Se and PPV of 96.4% and 97.6%, respectively, compared with Lead II. Detailed results for the normative and HF subject groups are shown in Table 2. The modified PT algorithm presented herein provided significant improvements in both Se and PPV as compared with the original PT algorithm for the bECG dataset.

The signal quality across subjects and population groups were very polarized, with 16 out of the 25 normative subjects having over 85% of the bECG waveforms pass the signal quality test, compared with 3 of the subjects having less than 40% of the bECG waveforms pass the signal quality test. The polarization of signal quality within the normative subjects is caused by a combination of low body weight (each of the three subjects weighed less than 61.4 kg) and a high impedance electrode/skin interface, which varies on a subject-by-subject basis.

**Table 1:** QRS classification using the original and modified Pan–Tompkins algorithm on standard databases.

Database	Total beats	Original PT^a^		Modified PT		
		Se^b^ (%)	PPV^c^ (%)	Se (%)	PPV (%)	SQI^d^ pass (%)
MITDB^e^	109,494	99.73	99.33	99.58	99.95	98.07
EDB^f^	790,565	99.71	95.48	99.87	99.80	96.55
Cumulative	900,059	99.72	95.93	99.83	99.82	96.73

^a^PT: Pan–Tompkins.

^b^Se: sensitivity.

^c^PPV: positive predictive value.

^d^SQI: signal quality index.

^e^MITDB: MIT-BIH database.

^f^EDB: European ST-T database.

**Table 2:** Buttocks electrocardiogram beat classification results comparing the original and modified Pan–Tompkins algorithm.

Study Cohort	Total beats	Modified PT^a^			Original PT	
		SQI^b^ pass (%)	Se^c^ (%)	PPV^d^ (%)	Se (%)	PPV (%)
Normative	30,075	79.6	98.2	98.1	86.1	77.7
HF^e^	41,820	45.4	94.2	96.6	62.2	59.6
Cumulative	71,895	60.1	96.4	96.7	72.2	67.4

^a^PT: Pan–Tompkins.

^b^SQI: signal quality index.

^c^Se: sensitivity.

^d^PPV: positive predictive value.

^e^HF: heart failure.

In addition, HF subject bECG signal quality was generally lower than that of the normative subjects (45.4% compared with 79.6%). The abnormal morphologies of the HF subjects’ bECG is a contributing factor to the lower percentage of sufficient quality regions. In addition, beats that result in an abnormal rhythm are not included when generating the ensemble average, resulting in a higher percentage of rejected regions for the HF subjects compared with normative.

Despite the challenges of using dry electrodes on a toilet seat, the percentage of acceptable waveforms, as well as the corresponding Se and PPV, are more than sufficient for accurate estimation of physiologic parameters. Instrumentation improvements focused on increasing the signal-to-noise ratio when using dry electrodes are expected to significantly increase the percent of waveforms that pass the signal quality algorithm.

### Cardiac Intervals Are Accurately Extracted From the Buttocks Electrocardiogram

**Figure 8:**
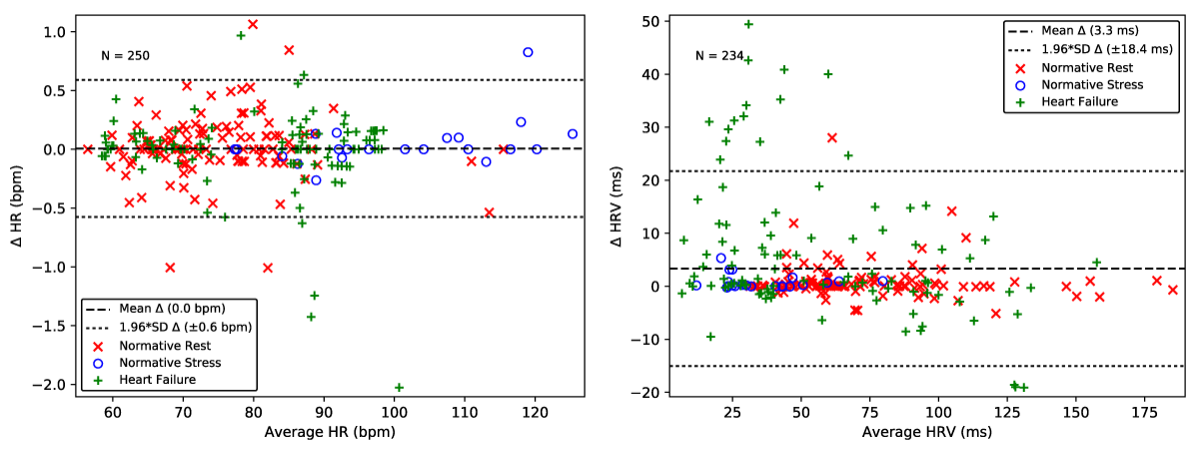
Heart rate (left) and heart rate variability (right) extracted from the bECG signal for normative rest (red x), normative post-stress (blue o), and heart failure (green +) are closely aligned with those extracted from the gold standard Lead II ECG. The dashed line shows the mean error. The dotted lines show 1.96 times the SD, corresponding to a 95% limits of agreement. bECG: buttocks electrocardiogram; ECG: electrocardiogram.

HR and HRV are calculated per recording for the bECG and Lead II ECG waveforms for all subjects at rest and post stress. A total of 250 recordings passed SQI and were analyzed for HR and 234 for HRV; results are shown as Bland-Altman plots in Figure 8. The automated delineation algorithms resulted in an excellent agreement between the bECG and the gold-standard HR, with virtually no bias and only six outliers having an error greater than 1 bpm. The SDDN HRV was clustered very close to the zero-error line, but with positive bias induced by a small number of significant outliers dominated by the HF population, shown in Figure 8. Although the results are excellent for a dry electrode system with no skin preparation, additional enhancements to the automated SQI algorithm may provide opportunities to further improve results for those with cardiovascular disease.

**Figure 9:**
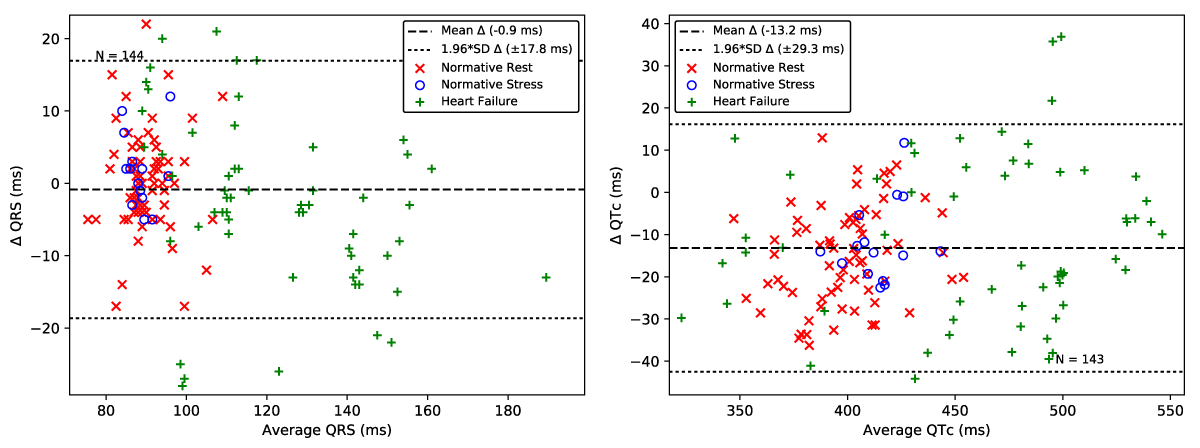
QRS duration (left) and QTc interval (right) extracted from the bECG signal for normative rest (red x), normative poststress (blue o), and heart failure (green +) are closely aligned with those extracted from the gold-standard Lead II ECG. The dashed line shows the mean error. The dotted lines show 1.96 times the SD, corresponding to a 95% limits of agreement. bECG: buttocks electrocardiogram; ECG: electrocardiogram; QTc: corrected QT.

The results comparing the bECG QRS duration and QT_c_ interval to Lead II are presented as Bland-Altman plots in Figure 9. A smaller number of recordings are included in the QRS duration and QT_c_ interval analysis compared with the HR and HRV analysis. This is due to the more stringent requirements imposed by the two additional beat rejection criteria when ensemble averaging and due to the rejection of recordings that did not have sufficiently clear features for delineation by the trained expert.

The Bland-Altman plot in Figure 9 shows a near zero bias in the QRS measures and 1.96 times SD of 17.8 ms. The Bland-Altman plot in Figure 9 shows a −13.2 ms bias in the QT_c_ interval, with an error of 29.3 ms (1.96 times the SD). The accuracy of the seated bECG measures of QRS duration and QT_c_ interval are within the limits of the expected accuracy of manual determination with a caliper, which has an error between 20 and 40 ms [52] and four standard automated approaches that have a 1.96 times SD error of over 35.5 ms [54,55]. Correlation analysis between the normative and HF subject groups showed statistically significant differences for bECG QRS duration (*P*<.001) and QT_c_ interval (*P*<.001) using the Student *t*-test. Algorithm refinement will provide an opportunity to remove outliers that have a significant impact on the overall SD, however, the present results compare well with the existing standards for measurement.

## Discussion

### Principal Findings

This study demonstrates that a dry electrode, toilet seat–based ECG provides robust determination of HR, HRV, QRS duration, and QT_c_ interval as compared with a standard Lead II ECG captured using traditional wet electrodes (Table 3). Results showed that the bECG was most closely correlated with standard Lead II, showing clinical relevance and demonstrating confidence in the fully integrated toilet seat measures. The success of the toilet seat–based ECG is attributed to the advanced signal processing algorithms presented herein, which have been custom designed for noisy, dry electrode ECG signals.

Standard algorithms designed for clinical grade devices expect a certain level of signal quality and would either perform poorly or reject too large of a percentage of recorded data for wearable and connected devices. To ensure that a high percentage of the waveform is not rejected, the signal quality rejection algorithm in this study was designed to reject only regions where the subsequent delineation algorithm will perform poorly. While providing robust results for the system it was designed for (ie, a nonstandard dry electrode ECG), these algorithms also excel at analyzing hospital-grade ECG signals without any modification, as demonstrated using the MITDB and EDB standard databases. The resulting Se and PPV are comparable with best-in-class algorithms that have been designed specifically for use with these databases, with exceptional accuracy on EDB, a much more challenging dataset for delineation algorithms.

### Limitations and Future Work

One limitation of this study is that data were recorded from subjects in either a lab or a clinical setting. While subjects were instructed to sit as they would at home during actual use, data captured in the home may result in additional motion artifacts and increase variability in signal quality. However, our results and the success of the signal quality algorithm suggest that in-home data can be successfully analyzed, even if a large percent of the signal does not have sufficient signal quality.

**Table 3:** Principal cardiac interval results of the buttocks electrocardiogram compared with Lead II for the normative and heart failure cohorts.

Study Cohort	HR^a^ (SD), in bpm	HRV^b^ (SD), in ms	QRS^c^, (SD), in ms	QT_c_^d^ (SD), in ms
Normative	−0.0 (0.3)	−1.0 (3.4)	−0.5 (6.6)	14.5 (11.1)
Heart failure	0.0 (0.3)	−6.6 (13.2)	2.9 (11.5)	11.2 (19.1)
Cumulative	0.0 (0.3)	−3.4 (9.4)	0.9 (9.1)	13.2 (15.0)

^a^HR: heart rate.

^b^HRV: heart rate variability.

^c^QRS: QRS duration.

^d^QT_c_: correct QT interval.

Similarly, this study did not investigate strain during use, which has the potential to modulate physiologic state in a similar fashion as the Valsalva maneuver. Future studies will investigate both the robustness of ECG measures during strain as well as the potential to detect cardiovascular shifts during strain that may have clinical value. Finally, the algorithms presented herein were only tested on ECG signals. To demonstrate broader applicability, future work will investigate the ability of the proposed approach to improve the performance of measurements captured with other signals such as the photoplethysmogram, which is commonly used in commercially available wearable technologies.

Despite these limitations, the results from this study lay the foundation for future studies on the clinical impact of the toilet seat measures, by having successfully demonstrated the accurate extraction of key cardiac intervals and parameters from the bECG as compared with a clinical gold-standard on both normative and HF populations. Since confidence in the form factor and measurements have been achieved, an in-home observation study and a subsequent intervention-based study can be executed. The initial in-home observational study will utilize daily HR, HRV, QT, and QRS measurements to create an alert-based system for HF patients. In the subsequent intervention study, cardiologists will be given the option to change medications or request a visit to the clinic based on automated alerts generated from seat data. This study will quantitatively determine how decision-making is affected through the enhanced monitoring capabilities of the seat, as well as how outcome events are impacted, such as hospitalization and the quantity of unnecessary procedures.

### Broad Impact

In addition to directly enabling the fully integrated toilet seat, the present algorithms have applicability to wearable and internet-connected in-home medical devices that generate a large amount of data and are used in an uncontrolled environment, where optimal sensor placement and consistent signal integrity cannot be guaranteed. The algorithms from this study are not computationally complex and have the potential to be executed by the on-board processors present in many wearable devices with minor modifications. By combining signal quality classification, accurate delineation, and robust ensemble averaging, new applications can be realized, such as cuffless blood pressure and noninvasive cardiac output monitoring. Utilizing this approach, additional sensors and measurements can be integrated into wearable and connected devices, creating novel comprehensive remote cardiovascular monitoring systems. Such devices have the potential to fill a gap in patient monitoring by capturing trend data that has been previously unattainable, through daily measurements and ensured compliance requiring no change in habit. This will enable new approaches and capabilities in the diagnosis and treatment of cardiovascular disease, including those with HF and hypertension. Through the successful development, deployment, and integration with clinical practice, wearable and connected medical devices that monitor clinically relevant measures can facilitate the transition from a reactive- to proactive-based approach to health care.

## Abbreviations

bECG: buttocks electrocardiogram
ECG: electrocardiogram
EDB: European ST-T database
FN: false negative
FP: false positive
HF: heart failure
HR: heart rate
HRV: heart rate variability
MIT-BIH: Massachusetts Institute of Technology - Beth Israel Hospital
MITDB: MIT-BIH database
PPV: positive predictive value
PT: Pan–Tompkins
QTc: corrected QT interval
RMS: root mean square
Se: sensitivity
SQI: signal quality index
TP: true positive
